# Improving osteoarticular characterization in magnetic resonance imaging: the role of simulated computed tomography sequences

**DOI:** 10.1590/0100-3984.2024.0048-en

**Published:** 2025-02-24

**Authors:** Gabriel Brito-Barbosa, Felipe Bortoloni Pires Correa, Leonor Garbin Savarese, Mateus Andrade Hernandes, Paulo Moraes Agnollitto, Marcelo Novelino Simão, Marcello Henrique Nogueira-Barbosa

**Affiliations:** 1 Department of Medical Imaging, Hematology, and Clinical Oncology, Faculdade de Medicina de Ribeirão Preto da Universidade de São Paulo (FMRP-USP), Ribeirão Preto, SP, Brazil

**Keywords:** Magnetic resonance imaging, Multidetector computed tomography, Bone and bones, Joints, Ressonância magnética, Tomografia computadorizada multidetectores, Osso e ossos, Articulações

## Abstract

Increasing tissue contrast for bone assessment on magnetic resonance imaging has been the aim of several recent studies, and various techniques have been proposed for that purpose, including ultrashort echo time sequences, zero echo time sequences, and gradient echo sequences in various acquisition forms. In this article, we discuss the fast field echo resembling a computed tomography using restricted echo-spacing (FRACTURE) sequence, which we have started to use routinely in our practice. The FRACTURE sequences are based on the acquisition of gradient echo sequences with different echo times and specific postprocessing. Gradient echo sequences are widely available on magnetic resonance imaging scanners, which is an advantage for the use of a FRACTURE sequence. However, being more susceptible to metal artifacts, a FRACTURE sequence is of limited utility in patients with metallic implants or medical devices. The aim of this article is to illustrate the use of FRACTURE sequences in various contexts, including osteoarticular infection, inflammatory arthropathy, bone tumors, fractures, and crystal deposition diseases.

## Abstract (pt)

Aumentar o contraste tecidual nas imagens obtidas com a ressonância magnética para avaliação óssea tem sido o objetivo de vários estudos recentes e diversas técnicas têm sido propostas para este fim, incluindo sequências com tempo de eco ultracurto, tempo de eco zero, além de sequências com eco de gradiente, em variadas formas de aquisição. Neste artigo trazemos o uso da sequência FRACTURE (acrônimo do inglês para *fast field echo resembling a CT using restricted echo spacing*), que passamos a utilizar rotineiramente no nosso serviço. Esta técnica se baseia na aquisição de sequência gradiente eco com diferentes tempos de eco e pós-processamento específico. As sequências gradiente eco são amplamente disponíveis nos equipamentos de ressonância magnética, o que representa uma vantagem para esta sequência. Contudo, por ser mais suscetível a artefatos metálicos, é limitada em pacientes com implantes e dispositivos metálicos. O objetivo deste artigo é ilustrar o uso da sequência FRACTURE em diferentes contextos, incluindo, entre outros, infecção osteoarticular, artropatia inflamatória, tumores ósseos, fraturas e doenças de deposição de cristais.

## INTRODUCTION

The role of magnetic resonance imaging (MRI) sequences that simulate the bone tissue contrast achieved with computed tomography (CT), known as MRI-based simulated CT (sCT) sequences, has been the object of recent studies^(1-3)^. The limited ability to characterize cortical and trabecular bone tissue is one of the weaknesses of MRI, and improving the tissue contrast for mineralized bone would be an important achievement. The MRI-based sCT sequences have great potential to aid in the diagnosis of inflammatory diseases, neoplasia, trauma, and anatomical variations. These sequences are useful in demonstrating bone fragmentation and resorption, as well as periosteal reaction, thus facilitating the identification and improving the understanding of deformities and of complex bone remodeling processes. The main MRI-based sCT sequences include ultrashort echo time (UTE), zero echo time (ZTE), and gradient echo susceptibility-weighted imaging (SWI) sequences, as well as volumetric multi-gradient echo Dixon (BoneMRI) sequences that employ deep learning for volumetric acquisition and volumetric gradient echo sequences with short in-phase echo times and short flip angles, designated 3D-Bone^(1,4-6)^.

**Figure 1:**
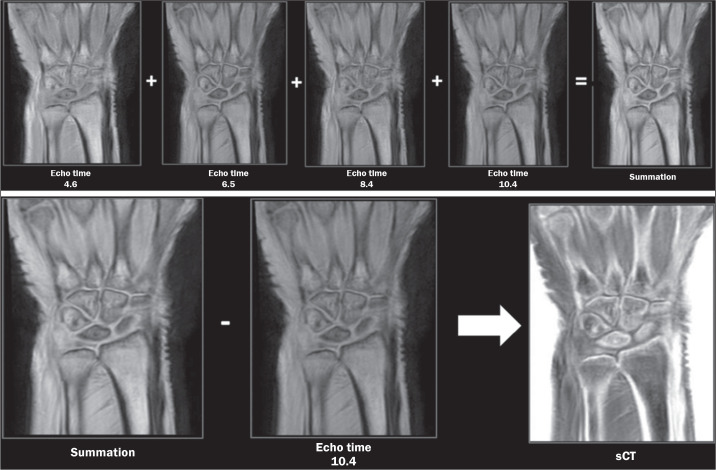
FRACTURE sequence MRI acquisition technique in which different echo times are obtained and summed. The last echo time is subtracted from the summation to form an image in which the gray scale is inverted, simulating the tissue contrast achieved with CT.

One currently available MRI-based sCT sequence is the fast field echo resembling a CT using restricted echo-spacing (FRACTURE) sequence, which is obtained by acquiring a gradient echo sequence with different echo times, followed by postprocessing. The first step in the postprocessing of FRACTURE sequences is the summation of all acquisitions with different echo times, which produces an image with a high signal-to-noise ratio. After summation, the image of the last echo pulse is subtracted from the summed image and the final product undergoes grayscale inversion, which produces an image with tissue contrast similar to that of CT^(7)^, as illustrated in Figure 1.

A FRACTURE sequence can be acquired in 1.5-T and 3.0-T scanners and, as described, uses different echo times for 1.5-T and 3.0-T scanners, respectively, that correspond to the in-phase acquisition times of 4.6 ms and 2.3 ms, with isotropic voxels of 0.62 mm and 0.7 mm, resulting in acquisition times of 4:56 and 6:48 min^(6)^. Johnson et al.^(7)^ described the acquisition of a FRACTURE sequence in a 3.0-T scanner, with a field of view of 160 × 160 mm. At our institution, FRACTURE sequences have been acquired in 1.5-T scanners, with the parameters described above. However, we have not acquired volumetric sequences and the images have been acquired with a slice thickness of 3.5 mm and an interslice gap of 1.75 mm, with a repetition time of 30 ms and a flip angle of 15°, resulting in an acquisition time of 4:18, the shoulder being used as an example.

The benefits of the FRACTURE sequence include its availability, because it is based on gradient echo sequences that are widely available on MRI scanners, high spatial resolution close to that of CT with a feasible examination time, the use of simple, minimal postprocessing, the non-use of ionizing radiation, and, finally, the potential to perform additional quantitative postprocessing techniques for research purposes^(7)^. Those additional quantitative postprocessing techniques include the assessment of the bone microstructure, the quantification of bone porosity as a biomarker of fracture risk, and the evaluation of the response to treatment in patients with osteoporosis or osteopenia^(8)^.

Despite its benefits, the FRACTURE sequence is highly susceptible to metal artifacts, as well as to motion artifacts. These limitations are more evident in FRACTURE sequence than in other MRI-based sCT sequences, such as UTE and ZTE sequences^(6)^.

The use of MRI-based sCT sequences should become a reality at musculoskeletal radiology centers. The objective of this pictorial essay is to illustrate and discuss the benefits of this technique for improving bone tissue contrast, particularly the usefulness of the FRACTURE sequence, on the basis of clinical cases from daily practice.

## OSTEOARTICULAR INFECTION

**Figure 2:**
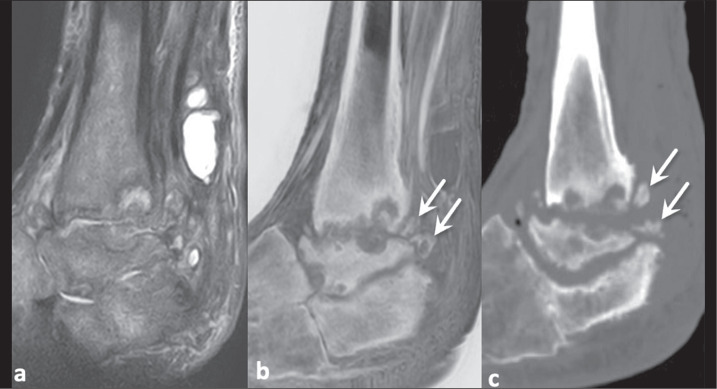
Neuropathic arthropathy and infection in a 63-year-old man with diabetes. Proton density-weighted MRI sequence with fat saturation (a) and FRACTURE sequence (b). Small detached bone fragments were not evident on conventional MRI, which was very useful for identifying the extensive inflammatory changes, but were visualized on the FRACTURE sequence (arrows in b) and confirmed on sagittal CT (arrows in c).

Conventional T1-weighted sequences and fluid-sensitive sequences are essential for characterizing the osteoarticular and periarticular infectious process, with bone marrow edema being a predictor of bone involvement. The use of MRI-based sCT sequences can increase the conspicuity of mineralized trabecular and cortical bone, thus improving the identification of deformities, as well as the evaluation of reactive bone sclerosis, bone resorption, and chronic bone fragmentation. In chronic osteomyelitis, for example, studies have demonstrated the role of a ZTE sequence in better characterizing cloacae and sequestra^(9,10)^. Figure 2 illustrates a case of septic arthritis and osteomyelitis in the ankle, in which a FRACTURE sequence complemented the evaluation by traditional sequences, facilitating the characterization of remodeling and bone resorption of the articular surfaces, reactive subchondral bone, and chronic bone fragmentation, changes confirmed by CT examination.

## INFLAMMATORY ARTHROPATHIES

In patients with spondyloarthritis, MRI is the imaging modality of choice for detecting sacroiliitis^(11-13)^. The use of MRI is important for detecting active inflammation and for characterizing the presence of postinflammatory structural damage, such as bone erosion, subchondral osteosclerosis, and partial or complete ankylosis^(14)^.

In our review of the literature, we did not identify any studies that evaluated the role of the FRACTURE sequence specifically in inflammatory arthropathies. However, in a study of patients clinically suspected of having inflammatory sacroiliitis, Jans et al.^(15)^ compared the performance of conventional T1-weighting with that of an MRI-based sCT sequence acquired in a manner similar to that employed to acquire a FRACTURE sequence (using multi-echo gradient echo pulse sequences) for the detection of erosions, sclerosis, and ankylosis of the sacroiliac joints. The difference between the sequence used in that study^(15)^ and a FRACTURE sequence is the postprocessing, which, in the sequence used by those authors, was performed with a deep learning technique that allowed even the measurement of attenuation (in HU) in the MRI-based sCT images. They found that the MRI-based sCT sequence accurately identified 94% of the erosions, compared with 86% for the conventional T1-weighting images that have been used as the sequence of choice for this evaluation. Interobserver and intraobserver reliability was comparable to that of CT^(15)^.

**Figure 3:**
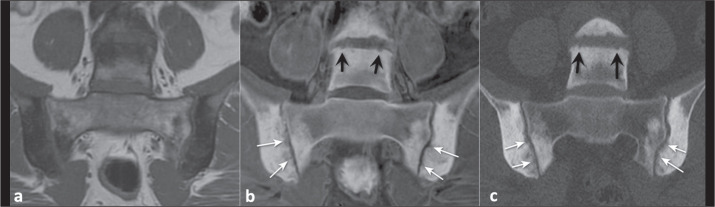
A 42-year-old woman with low back pain suspected of being due to axial spondyloarthritis. T1-weighted MRI of the sacroiliac joints (a), showing multiple, coalescent erosions together with subchondral sclerosis. The bony erosions (white arrows) and irregularity of the vertebral plateaus (black arrows) are best demonstrated on a FRACTURE sequence (b), findings that were confirmed on CT (c).

In the case of rheumatoid arthritis, MRI is also used to assess disease activity and structural damage, findings that can be objectively classified in the rheumatoid arthritis MRI scoring system proposed by the Outcome Measures in Rheumatology (OMERACT) group^(16)^. The detection of erosions in rheumatoid arthritis is known to be important because it contributes to the diagnosis and prognosis^(17)^. It is clear, then, that the detection of bone erosions is at the core of the diagnosis of inflammatory arthropathies, and that MRI-based sCT sequences have strong potential to contribute to the detection of these abnormalities^(14)^, as depicted in Figure 3.

## BONE TUMORS

**Figure 4:**
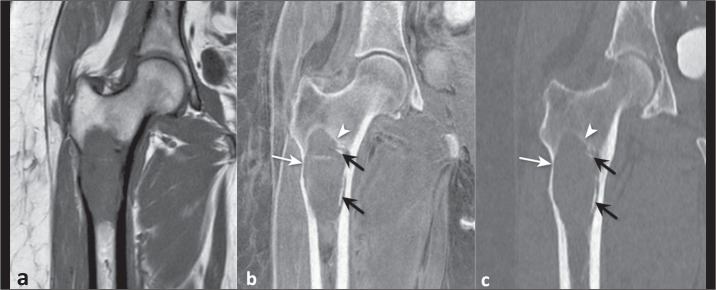
A 57-year-old woman with an expansile osteolytic lesion in the subtrochanteric region. In comparison with a T1-weighted MRI sequence (a), a FRACTURE sequence (b) better demonstrated a discrete marginal bone reaction (arrowhead), endosteal indentation (white arrow). and small calcified areas within the lesion (black arrows), in accordance with coronal reformatting of a CT image (c).

The bone destruction pattern, periosteal reaction, and lesion contours are used in order to predict the degree of tumor aggressiveness by conventional radiography^(18)^. In addition, characterization of the pattern of bone matrix mineralization can help predict the histology^(19)^. Currently, conventional radiography continues to be the imaging modality of choice for the initial investigation of bone tumors, as recommended by the American College of Radiology^(20)^. In a retrospective study of 32 patients, Gersing et al.^(21)^ evaluated the agreement between conventional X-rays and MRI-based sCT sequences in combination with simulated X-rays. The simulated X-rays were also derived from volumetric MRI sequences acquired on 3.0-T scanners with postprocessed T1-weighted gradient echo pulse sequences. The authors evaluated the degree of tumor aggressiveness, including the pattern of bone destruction and periosteal reaction, and found good agreement between the MRI-based sCT sequence and the conventional X-rays. Similarly, Xu et al.^(22)^ demonstrated comparability between the findings of ZTE sequences and those of CT in the evaluation of bone tumors. To our knowledge, there have been no studies specifically evaluating the role of a FRACTURE sequence in this context. Adding MRI-based sCT sequences to routine protocols for the investigation of bone neoplasms may become essential to aid in the evaluation of tumor aggressiveness, the pattern of involvement, and local staging (Figure 4).

## FRACTURES

**Figure 5:**
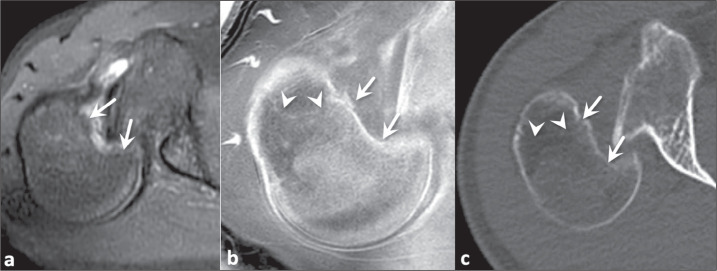
A 19-year-old man with chronic posterior subluxation of the right shoulder and chronic impacted fracture of the humeral head. A reverse Hill-Sachs lesion (arrows) was well demonstrated in the different sequences acquired, including a proton density-weighted MRI sequence with fat saturation (a). However, a FRACTURE sequence (b), like a CT image (c), allowed better evaluation of the cortical bone tissue in the impacted fracture of the humeral head (arrowheads in b and c), because of its characteristics of chronicity and absence of bone edema.

The assessment of bone fracture lines and margins is one of the main advantages of MRI-based sCT sequences (Figure 5). In the knee, for example, an MRI-based sCT sequence is capable of identifying the fracture, and in cases of anterior cruciate ligament avulsion, it provides accurate measurement of the avulsed fragment and the degree of retraction, aiding in surgical planning^(21)^.

Spinal and hip fractures increase the risk of death for five and ten years after the event, respectively, which makes their identification of fundamental importance. In a study involving 30 patients, Schwaiger et al.^(23)^ evaluated the performance of MRI-based sCT sequences, in particular the three-dimensional T1-weighted spoiled gradient echo (T1SGRE) and UTE sequences, and those sequences showed good agreement with conventional CT in detecting fractures and degenerative changes in the spine, especially the T1SGRE sequence, which was shown to be more robust than the UTE sequence, presenting superior sensitivity, specificity, and accuracy for detecting fractures. According to the authors, in the T1SGRE sequence, spinal fractures could be measured and grouped in accordance with the main classification systems available, and the measurements obtained with this sequence were practically identical to those obtained with conventional CT.

In the context of head trauma caused by gunshot, Gascho et al.^(24)^ found equivalence between CT and FRACTURE sequences in terms of the findings of bone damage and fractures, with both showing performance superior to that of conventional MRI sequences. In cases of shoulder dislocation with Hill-Sachs lesion (Figure 5), one challenge is measurement of the bone defect, which usually presents an oblique course that does not follow the path of the MRI acquisition planes. In that context, Cui et al.^(25)^ recently found equivalent performance between the volumetric FRACTURE sequence and CT in quantifying bone loss, as well as in measuring morphological parameters of the shoulder. In another recent study, Feuerriegel et al.^(26)^ evaluated the role of MRI-based sCT sequences in the context of shoulder trauma by evaluating the performance of different techniques that simulate CT tissue contrast, using conventional CT as the reference standard. The authors found that T1-weighted volumetric gradient echo sequences, UTE sequences, and FRACTURE sequences showed similar accuracy for identifying glenoid bone loss in bony Bankart lesions.

## CRYSTAL DEPOSITION DISEASES

**Figure 6:**
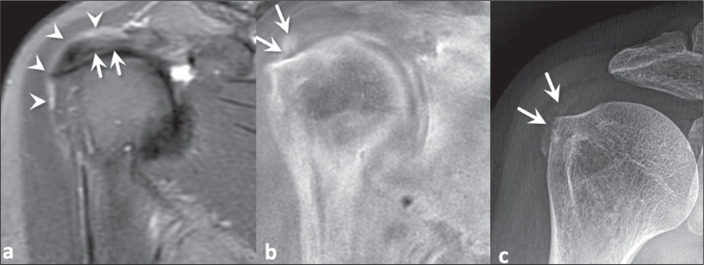
A 49-year-old woman complaining of severe shoulder pain. Conventional T2-weighted MRI sequence with fat saturation (a), showing supraspinatus tendinopathy (arrows) and subacromial subdeltoid bursitis (arrowheads). One of the main causes of shoulder pain is calcifications resulting from crystal deposition diseases, and in conventional sequences these calcifications present low signal intensity and might be misidentified, as in this case. A FRACTURE sequence showed a focus suspicious for calcification (arrows in b), confirmed on a conventional X-ray (arrows in c).

Crystal deposition diseases are common, especially in the elderly population^(27,28)^. Although asymptomatic in some cases, they can eventually become symptomatic and a cause for concern. The most common crystals deposited include calcium pyrophosphate dihydrate, calcium hydroxyapatite, and monosodium urate, the last causing the disease known as gout. The deposition of calcium hydroxyapatite is predominantly periarticular, in tendons and bursae, and is a source of periarthritis, especially in the resorptive phase^(29)^. The joint most often affected is the shoulder (Figure 6), followed by the hip. Among patients with shoulder pain, calcific tendinopathy of the rotator cuff has an estimated prevalence of 6.8-54.0%^(30,31)^. In 2015, Nörenberg et al.^(32)^ published a study showing that the addition of SWI sequences to the routine shoulder protocol increased the detection of calcifications when conventional radiography was used as the reference standard. In calcium pyrophosphate deposition disease, calcium pyrophosphate dihydrate crystals can occur in cartilage (chondrocalcinosis) and fibrocartilage, such as the menisci. In a study published in 2019, Finkenstaedt et al.^(33)^ showed that UTE sequences were able to demonstrate deposits of calcium pyrophosphate crystals in the menisci that, preliminarily, were concentrated mainly in the avascular zones. Although ZTE sequences are useful for demonstrating punch erosions and calcified foci in gouty tophi, the benefit of these sequences in this context is not yet fully clear^(9)^.

## BENEFITS OF THE FRACTURE SEQUENCE IN OTHER CONTEXTS

**Figure 7:**
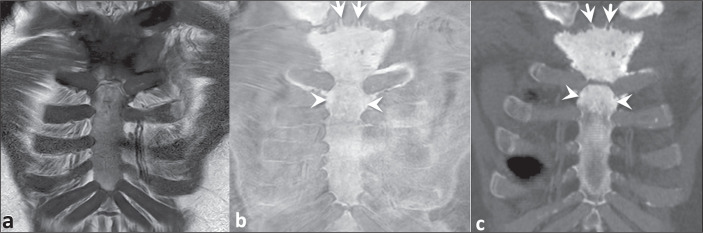
A 56-year-old man with SAPHO syndrome. A T1-weighted MRI sequence (a) showing markedly low signal intensity adjacent to the sternoclavicular joints, indicating the presence of osteitis. A FRACTURE sequence (b) providing greater detail on the bone irregularities with the formation of small areas of hyperostosis (arrows), a finding confirmed on CT (c). Note also the osteosclerosis in the sternal manubrium, which is more evident on the FRACTURE sequence than on the conventional T1-weighted sequence (arrowheads).

The FRACTURE sequence has been shown to be useful for characterizing erosion on articular surfaces, identifying bone sclerosis in patients with osteitis and bone hyperostosis in patients with synovitis, acne, pustulosis, hyperostosis, and osteitis (SAPHO) syndrome (Figure 7). These findings are important for characterizing the disease and complement the ability of routine sequences, which demonstrate bone edema and synovitis.

**Figure 8:**
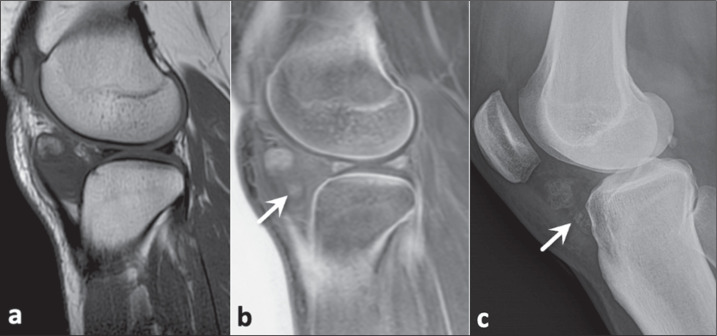
A 39-year-old woman with a chondroma in Hoffa’s fat pad, also known as the infrapatellar fat pad, confirmed by histopathology. In comparison with a conventional MRI sequence (a), the FRACTURE sequence (b) better identified the ossifications of the chondroma (arrows). A conventional X-ray (c) confirmed the presence of areas of ossification within the lesion.

**Figure 9:**
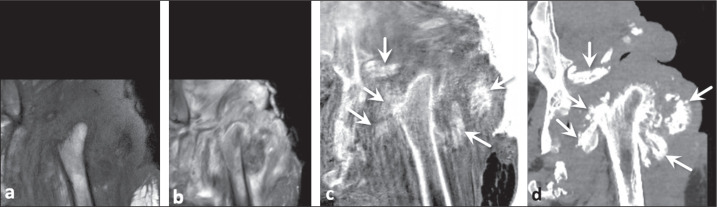
A 29-year-old man with neurogenic heterotopic ossifications secondary to a spinal cord injury. Inflammatory or infectious changes in the periarticular soft tissues of the left hip were best visualized on a conventional T1-weighted MRI sequence (a) and a STIR sequence (b). However, the chronic heterotopic soft tissue ossifications (arrows) were best demonstrated on a FRACTURE sequence (c), as confirmed on CT (d).

In soft tissues, calcifications in tumors such as chondromas (Figure 8) can be clearly visualized in FRACTURE sequences, as can heterotopic ossifications, including those of neurogenic origin (Figure 9), which are one of the most common orthopedic complications in the context of spinal cord injuries^(34)^.

**Figure 10:**
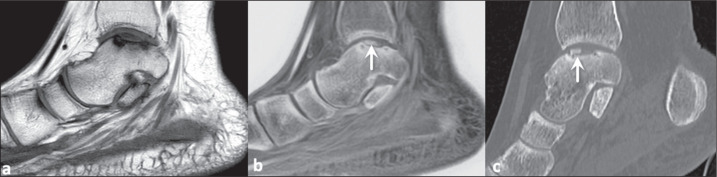
A 25-year-old woman with an osteochondral lesion in the talus. A T1-weighted MRI sequence (a) showing an unstable fragment detached from the talar dome. A FRACTURE sequence (b) more clearly identifying the bone fragment (arrow), a finding later confirmed on CT (c).

**Figure 11:**
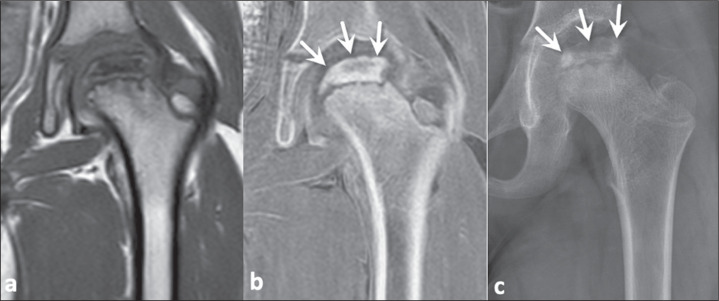
An 8-year-old boy with Legg-Calvé-Perthes disease. T1-weighted MRI sequence (a), FRACTURE sequence (b), and conventional X-ray (c), demonstrating avascular necrosis of the femoral head, characterized by bone resorption, fragmentation, and sclerosis in the femoral epiphysis (arrows). However, the finding was more visible on the FRACTURE sequence and the conventional X-ray.

**Figure 12:**
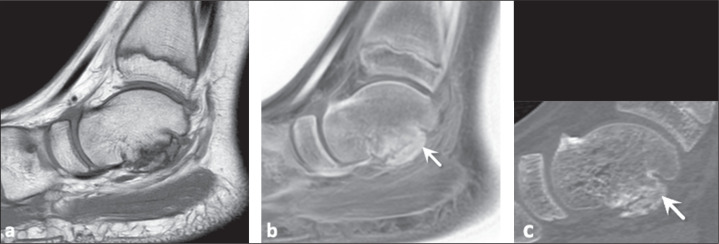
A 12-year-old boy with talocalcaneal coalition. Conventional T1-weighted MRI sequence (a) showing bony irregularities in the subtalar joint, with no evidence of bony bridges, suggesting fibrous union. However, bony bridges consistent with a talocalcaneal bony bar (arrows) were identified on a FRACTURE sequence (b) and on a CT image reformatted in the sagittal plane (c).

Osteochondral lesions may include defects of articular cartilage and subchondral bone. Although various classifications have been proposed, all define the most advanced grade as when the bone fragment is detached, which has practical implications for treatment^(35,36)^, a finding that can be visualized well on FRACTURE sequences (Figure 10). In addition, the findings of avascular necrosis of the femoral head and of Legg-Calvé-Perthes disease, such as bone sclerosis, remodeling, and fragmentation, tend to be more conspicuous on FRACTURE sequences (Figure 11). In cases of bone union (Figure 12), a FRACTURE sequence allows adequate identification of bone fusion and helps in differentiating among the types of union.

## CONCLUSION

The use of MRI-based sCT sequences has been shown to be beneficial in the evaluation of osteoarticular tissues in various clinical contexts. As was our objective, we have illustrated the different situations in which a FRACTURE sequence provided additional information that complemented the information from routine sequences, findings confirmed in correlation with X-rays or CT images. However, we emphasize that studies on MRI-based sCT sequences are still relatively scarce, particularly when we look for studies that compare the diagnostic performances of the different types of such sequences.
